# Design and Characterization of a High Resolution Microfluidic Heat Flux Sensor with Thermal Modulation

**DOI:** 10.3390/s100706594

**Published:** 2010-07-09

**Authors:** Sung-Ki Nam, Jung-Kyun Kim, Sung-Cheon Cho, Sun-Kyu Lee

**Affiliations:** Department of Mechatronics, Gwangju Institute of Science and Technology, Oryong-dong, Buk-gu, Gwangju 500–712, Korea; E-Mails: sknam@gist.ac.kr (S.-K.N.); junggyun@gist.ac.kr (J.-K.K.); csc33033@gist.ac.kr (S.-C.C.)

**Keywords:** heat flux sensor, thermopile, electro-thermal domain model, microfluidic application

## Abstract

A complementary metal-oxide semiconductor-compatible process was used in the design and fabrication of a suspended membrane microfluidic heat flux sensor with a thermopile for the purpose of measuring the heat flow rate. The combination of a thirty-junction gold and nickel thermoelectric sensor with an ultralow noise preamplifier, a low pass filter, and a lock-in amplifier can yield a resolution 20 nW with a sensitivity of 461 V/W. The thermal modulation method is used to eliminate low-frequency noise from the sensor output, and various amounts of fluidic heat were applied to the sensor to investigate its suitability for microfluidic applications. For sensor design and analysis of signal output, a method of modeling and simulating electro-thermal behavior in a microfluidic heat flux sensor with an integrated electronic circuit is presented and validated. The electro-thermal domain model was constructed by using system dynamics, particularly the bond graph. The electro-thermal domain system model in which the thermal and the electrical domains are coupled expresses the heat generation of samples and converts thermal input to electrical output. The proposed electro-thermal domain system model is in good agreement with the measured output voltage response in both the transient and the steady state.

## 1. Introduction

Heat flux (or heat transfer per unit area) and temperature are important boundary conditions of heat transfer problems. Various measurement techniques have been developed for precise analysis of the heat transfer problems. Of these techniques, heat flux measurement is a hot issue in many sensor application fields because it is directly related to the quantity of heat transferred [1].

High-precision thermometers for measuring the amount of heat flux are used in many applications: Eminoglu *et al*. [2] suggested infrared detectors with a suspended n-well resistor, which were implemented in a 0.8 μm CMOS process, Matsumiya *et al*. [3] and Baciocchi *et al.* [4] developed thermoelectric gas sensors using a thermo-electric thin-film of nickel oxide with a platinum catalyst thin-film on half of its surface Pt/NiO/alumina. Buchner *et al.* [5] and Kim *et al.* [6] proposed thermal flow sensors using thermopiles made of p-doped polysilicon and titanium–tungsten (WTi) with a nitride membrane, Hopper *et al.* [7] fabricated an IR surface temperature sensor and thermal flow sensor using CMOS and MEMS process with a SOI silicon oxide membrane. Johannessen *et al*. [8] demonstrated nanocalorimeter suspended membrane sensor for pL volumes of aqueous media fabricated by bulk silicon micromachining using anisotropic wet etching and photo and electron beam lithographic techniques. Liliana *et al.* [9] suggested an isoperibolic calorimeter to detect the exothermic and endothermic effects of the cell. Kao *et al.* [10] proposed a thermoelectric micro generator which has 24 thermocouples with suspended membrane.

A thermal modulation method is employed to detect the objective signal using internal heater without disturbance noise which comes from not only external noise, but also sensor itself. Moreover, a dynamic simulation method is developed for characterizing the fabricated heat flux sensor including electrical surroundings. This sensor can measure heat generated from live cells. It provides direct information about the physiological state of organisms and is helpful for the recognition of metabolic pathways [11]. Furthermore, a label-free detection of enzyme activities is possible. The heat flux sensor is calibrated by using a thermal modulation method to reduce low-frequency contact noise [12] from the signal output. The resolution and sensitivity of the sensor is identified and measured with various amounts of fluidic heat so as to investigate the suitability of the sensor for microfluidic application. The system model includes the electro-thermal behavior of a sensor with an integrated electronic circuit. The model is presented for the purpose of designing a micro heat flux sensor system and for analysis of the signal output. The electro-thermal domain system model of the microfluidic heat flux sensor is based on a bond graph methodology. The proposed system model is validated with measured data in calibration experiments and fluidic application environment.

## 2. Heat Flux Sensor Design

### 2.1. Heat flux sensor principle

**Figure 1.**
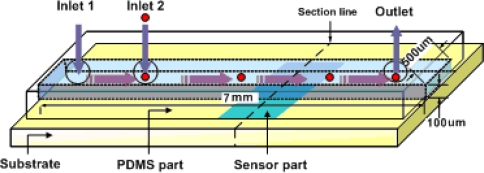
Schematic of the microfluidic heat flux sensor.

Figure 1 shows a schematic diagram of a micro heat flux sensor with a fluidic channel. A culture medium injected through the inlet 1 and this flow indicates reference value. The cells flow through the inlet 2 with culture medium and this flow indicates objective value. The objective heat flux is measured on the sensor part while an objective fluid flows through the polydimethylsiloxane (PDMS) fluid channel.

For this study, we used a CMOS process to fabricate a heat flux sensor composed of a thermopile structure. A thermopile can reduce the external power noise and self-heating problems caused by temperature dependent of resistance. The thermopile, which is based on the Seebeck effect, measures the temperature difference between two different points [13]. In other word, the temperature difference can be calculated using sensor output. Assuming thermal conductivity is a constant value because of small temperature differences, and temperature distribution between hot and cold junction is linear, heat flux passing through it can be estimated. Heat flux can be calculated from the Seebeck effect and Fourier’s heat conduction law:

(1) $$V_{\mathrm{AB}}=N\cdot \alpha_{\mathrm{AB}}\cdot \mathrm{dT}$$

(2) $$q\prime \prime =-k\frac{\mathrm{dT}}{\mathrm{dx}}=\frac{k\cdot V_{\mathrm{AB}}}{N\cdot \alpha_{\mathrm{AB}}\cdot l}$$

where q″ is conducted heat flux, k is thermal conductivity, N is the number of thermocouples in series, α_AB_ is Seebeck coefficient difference, *l* is the length of the thermocouples, and V_AB_ is the voltage output from thermoelectric effect.

**Figure 2.**
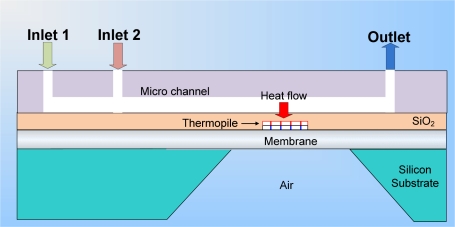
Lengthwise cross section view.

**Figure 3.**
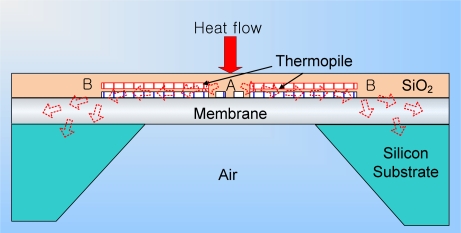
Cross section of sensor part.

Figures 2 and 3 show a cross sectional schematic of the heat flux sensor. When a heat flux passes through the membrane, the thermoelectric potential generated by the thermopile is proportional to the temperature difference between junction A and junction B. A micro heater can provide a calculated heat power to the sensor and sensitivity which is the ratio of sensor output voltage to heat power can be obtained. Using this sensitivity value, the heat flux can be obtained from the sensor output voltage. The output potential can be increased by increasing the number of thermocouple series. The membrane is suspended in air for the thermal isolation of junction A and supported at its edge (junction B) by a bulk micromachined silicon substrate, which acts as a thermal sink. The 0.2 μm thickness of the two metal thermocouple layers and the membrane is minimized to reduce their thermal mass. More accurate measurements can be obtained by increasing the thickness of the metal layers and the temperature difference between junction A and junction B. When the thickness of the metal layer is decreased, the sensor response time becomes smaller, but the generated thermoelectric output becomes smaller [14,15].

### 2.2. Fabrication process

**Figure 4.**
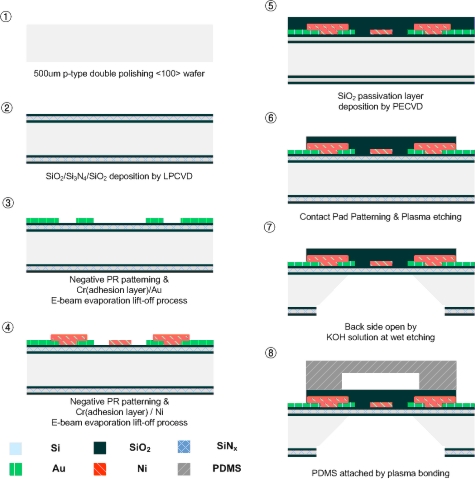
Fabrication steps for the microfluidic heat flux sensor.

Figure 4 shows the fabrication steps of the sensor. The process starts with a 101.6 mm diameter, 500 μm thick, double-side polished, *p*-type <100> silicon wafer. A 0.8 μm thick silicon dioxide (SiO_2_) layer, followed by a 0.4 μm thick silicon nitride (SiN_x_) layer on both sides on the silicon wafer, was deposited to compensate the stress on the silicon wafer. The next layer is a 0.2 μm thick layer of Au and Ni, which is patterned and etched by a lift-off process to define the thermopile and the calibration heater. A 0.4 μm thick layer of SiO_2_ is then deposited for electrical insulation between the Au and Ni. The SiO_2_ layer is patterned and etched to provide the junction of the thermopile.

Next is a 0.4 μm low stress layer of SiO_2_ as a passivation layer, which is subsequently patterned and etched to provide electric pads for the output terminals. The layers on the back of the silicon are opened for a wet etching process. To open the layers, we first pattern the wafer on the back of the substrate by using double-sided alignment and then do the etching. The wafer is immersed in a KOH solution at 80 °C. As a result of the back side wet etching process, a 700 × 700 μm^2^ area, 1.6 μm thickness of dielectric and transparent membrane is formed. The PDMS fluid channel is attached by plasma bonding.

**Figure 5.**
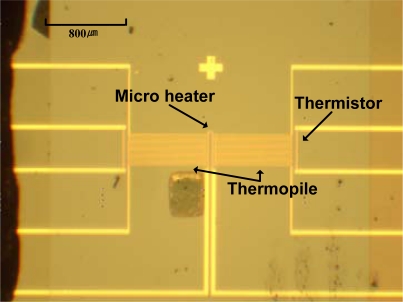
Micrograph of the top of the completed sensor.

Figure 5 shows a top view of the completed heat flux sensor. The thermopile and the micro calibration heater are located on top of a thermally and electrically suspended membrane. The thermopile is made of Au and Ni and consists of thirty thermocouples connected in series. The hot junctions are on the center of the membrane, whereas the cold junctions are on the silicon substrate. Due to its large thermal mass and good thermal conductivity, the silicon substrate works as a heat sink and keeps the cold junction at a steady state during the measurement. The thermistor is at the end of the cold junctions to keep the reference temperature constant.

## 3. Experiments

### 3.1. Thermal modulation

**Figure 6.**
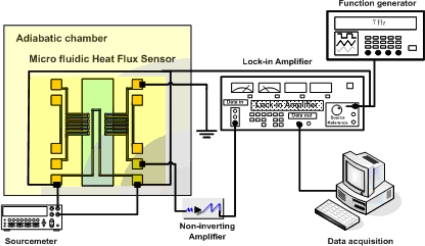
Schematic of the experimental setup.

The fabricated sensor is characterized with a micro calibration heater. To enhance the performance of the developed sensor, reducing under 1 Hz of low frequency noise in the output signal is needed. In this study, there is a thermal modulation a method is introduced to eliminate the low-frequency noise such as contact noise induced by imperfect contact between pairs of thermocouples and external thermal fluctuations. Figure 6 shows a schematic of the experimental setup for characterizing of the sensor.

To minimize the effect of external thermal fluctuations, the sensor was placed inside an insulation chamber, the temperature of which can be controlled to an accuracy level of 0.1 K. A sourcemeter supplies the power to the micro calibration heater. A function generator is connected to a lock-in amplifier and the heater for the reference input signal. The output voltage from the sensor is amplified with a non-inverting amplifier and connected to the signal input of the lock-in amplifier. Finally, the output signal from the lock-in amplifier is converted with a data acquisition module. The lock-in amplifier mixes the reference input signal and the sensor output signal together, and the results can be expressed as:

(3) $$V_{\mathrm{out}}=[V_{\mathrm{DC}}+V_{\omega }\text{sin}(\omega t+\phi_{\omega })+V_{2\omega }\text{sin}(2\omega t+\phi_{2\omega })]\cdot [V_{\mathrm{ref}\_\mathrm{DC}}+V_{\mathrm{ref}}\text{sin}(\omega t)].$$

The output then passes to a low-pass filter, which removes the *ωt* component, leaving the output for the lock-in amplifier. The results can be expressed as follows:

(4) $$V_{\mathrm{out}}=V_{\mathrm{DC}}(V_{\mathrm{ref}\_\mathrm{DC}}-V_{\mathrm{ref}})+\frac{1}{2}V_{\omega }V_{\mathrm{ref}}\text{sin}\phi_{\omega }+V_{2\omega }V_{\mathrm{ref}}\text{sin}\phi_{2\omega }$$

The output signal from the lock-in amplifier is a DC signal that is proportional to the magnitude of the input signal from the sensor and proportional to the sine of the angle, φ.

### 3.2. Sensitivity and resolution

**Figure 7.**
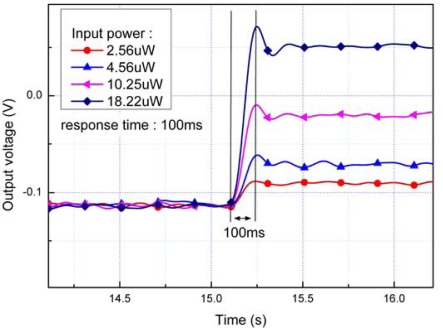
Response time of the sensor using various heat fluxes.

To calibrate the sensor and measure its sensitivity, the supplied power to the heater is increased in a stepwise manner and measured the steady state output voltage of the sensor. Table 1 shows a summary of the measurement conditions of the modulation. Figure 7 shows the time response of sensor for the various step input and it has 100 ms of response time.

**Figure 8.**
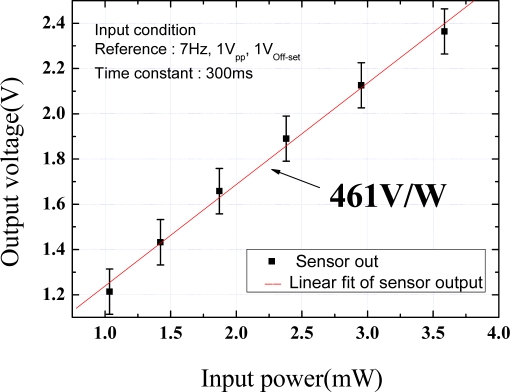
Calibration of the measured heat flux *versus* sensor output voltage.

Although the sensor response time is related to contact resistance, sensor geometry, and properties of sensor itself, but electrical circuit arrangement is the most important parameter. Heater modulation and lock-in amplifier system make response time late due to time constant of filter system. Moreover, time constant of electrical circuit and reduction of low frequency noise have trade off relations, so choosing proper time constant of modulation system and designing of heater for modulation is required for transient studies. The measured results plotted in Figure 8 show a very good linearity when the supplied power is in the 1 mW to 3 mW range. The sensitivity of the sensor, which is calculated by dividing the output voltage by the heat flux or the slope of the plot, is 461 V/W.

**Figure 9.**
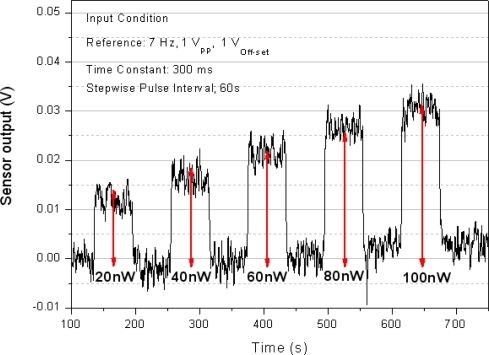
Resolution of the microfluidic heat flux sensor with the measured sensor output voltage and the input power of the heater.

Figure 9 shows that the sensor had a resolution of 20 nW and an output noise of 4 mV when the sensor output voltage was measured while the power supplied to the heater was increased from 20 nW to 100 nW.

The sensitivity results compare very favorably with other microfabricated thermopiles reported in the literature, such as Au/p-poly couples (0.94 V/W) [16], Bisb/Sb (4–6 V/W) [17], and GaAs/AlGaAs (145 V/W) [18]. This sensor also shows a much better resolution than the Bisb/Sb (100 nW) [17] and Ni/Au (25 nW) [19]. Kemp and Guan stated that the range of heat flux for a mammalian cell is from 0.01 to 329 pW per cell [20]. Mammalian red blood cells are typically smaller than 10 μm [21]. The measurement area (100 μm × 500 μm) of the developed sensor can contain a maximum of 5,000 cells. Depending on the number of cells, the required resolution of the sensor is therefore in the range of 50 nW to 1.6 μW.

### 3.3. Fluidic experiments

**Figure 10.**
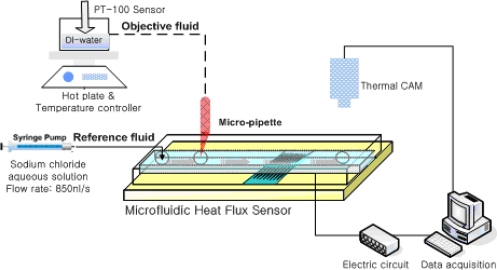
Schematic of the microfluidic experimental setup.

The heat flux sensor was tested with various amount of fluidic heat so as investigate its suitability in microfluidic application. Figure 10 shows a schematic of the experimental setup with a syringe pump, a hot plate, and an IR thermometer

**Figure 11.**
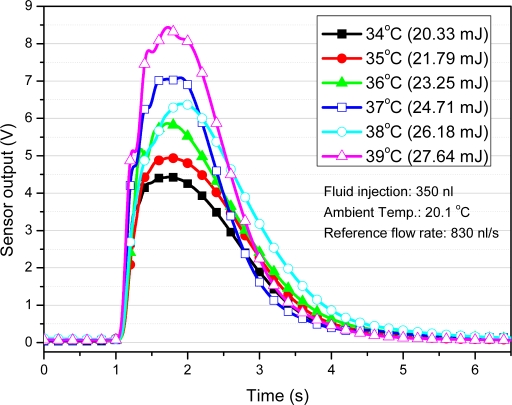
Sensor output with various amounts of heat from the fluid input.

**Figure 12.**
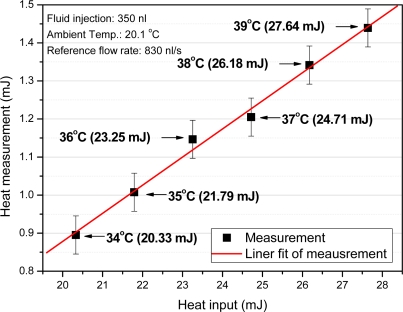
Heat power and different voltage output with various amounts of heat from the fluid input.

A sodium chloride aqueous solution is inserted as the reference fluid into a PDMS fluid channel with a width of 500 μm, a height of 100 μm, and a length of 7 mm. The flow rate is controlled by means of a syringe injection pump. The amount of heat from DI-water, which is the objective fluid, is controlled by a hot plate. The DI water is inserted via a micro pipette, which can control the volume of the fluid in the range of 0.1 to 100 μL. The inserted volume of fluid is 350 nL. Depending on the temperature, the whole heat of the inserted fluid varied from 14.48 to 27.64 mJ (from 41.37 to 78.97 J/g). Figure 11 shows the microfluidic heat flux sensor output response when the object passes steadily through the PDMS fluid channel. PDMS has very low thermal conductivity of 0.15 W/m k and insulates fluid from ambient convection. Furthermore, temperature of the inlet and the outlet water was monitored by thermal camera. Figure 12 shows the heat as calculated by the integration of the measured sensor output response along with the inserted amount of heat. It shows the linearity between the measured sensor output response and the inserted amount of fluidic heat.

The calculated amount of heat is only 4–5% of the actual inserted amount of heat. As a certain amount of heat from the reference fluid flows into the outlet of the PDMS fluid channel or into the exterior for the sensor structure components, the developed sensor measures only a limited amount of heat. Although the above-mentioned process has a minimum required resolution of 50 nW to 1.6 μW, the actual required resolution varies from 2.5 nW to 80 nW for a microfluidic application because heat is lost during the measurement process. Hence, thermal insulation between the PDMS fluid channel and the sensor is necessary to increase the performance of the sensor in microfluidic application.

## 4. System Modeling

### 4.1. Bond graph methodology

A system model is presented for the purpose of designing a micro heat flux sensor system. The model is based on the electro-thermal behavior of a sensor with an integrated electronic circuit. The signal output is also analyzed. An electro-thermal domain system model for the microfluidic heat flux sensor was constructed by means of a bond graph methodology.

Bond graph modeling is an attractive means of modeling a dynamic physical system [21,22]. It uses a language based on power exchange within a system model where the structure of the system model is shown graphically [23]. One of the most interesting advantages of bond graph modeling is that it can efficiently represent analytical models graphically in complicated cases involving the coupling of multiple energy domains (*i.e.,* mechanical, electrical, hydraulic, thermal, or magnetic domains) [24–28].

### 4.2. Thermal domain modeling

Conventionally, a thermal system is analogous to electricity: that is, temperature is similar to voltage and the heat flow rate is similar to a current. A thermal domain system can therefore be represented by thermal resistance and thermal capacitance in a thermal network. The model was developed using the commercial program 20-Sim available through Controllab B.V. [29].

**Figure 13.**
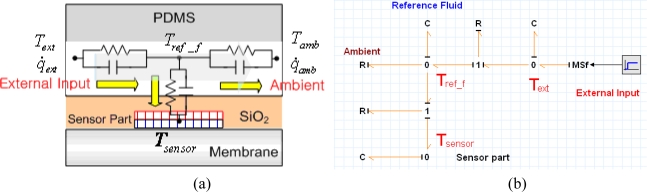
(a) Heat flow model of a fluid channel; (b) bond graph model of a fluid channel.

**Figure 14.**
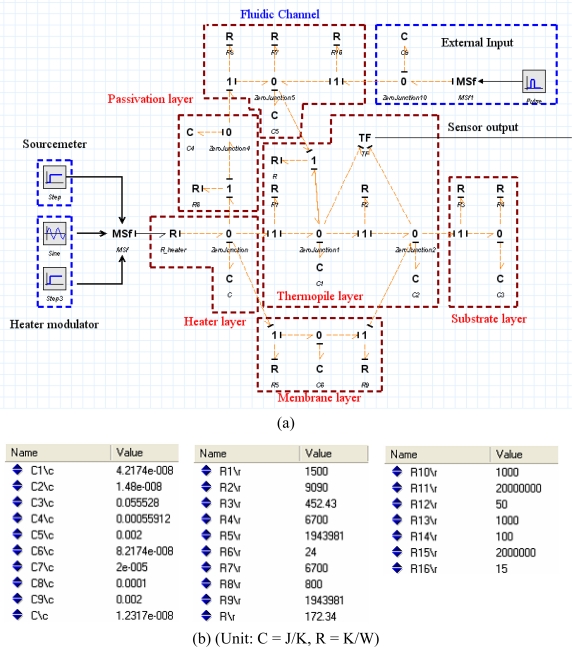
Bond graph model of (a) a heat flux sensor part and (b) values.

Figure 13(a) shows an equivalent thermal network model of the heat flow inside of the fluid channel based on 2D transient modeling; Figure 13(b) shows a bond graph model of the fluid channel part. The heat from the objective fluid flow inside the fluid channel and some of heat that flows to the developed sensor part and elsewhere flows simultaneously to the ambient. Heat loss caused by radiation is not considered due to the low temperature of fluid. Heat loss caused by convection and conduction can affect the result, so thermal resistance can be obtained from the real experiments. If the temperature between fluid on sensitive area and sensor surface is acquired, thermal resistance containing both convection and conduction can be calculated. However, thermal resistance and heat capacity of sensor part can be calculated from its material properties and geometry [30]. Figure 14 shows the bond graph model of the sensor part and values. The heat generated by the heater flows to the fluid channel through the passivation layer and the thermopile structure. The heat generated from the fluid channel flows to the thermopile structure and to the micro calibration heater. The sensor modeling part consists of a micro calibration heater for characterization of the sensor, a thermopile structure for measurement, a fluid channel model for microfluidic application, and a sourcemeter and the heater modulator for the thermal modulation method.

### 4.3. Electrical domain modeling

**Figure 15.**
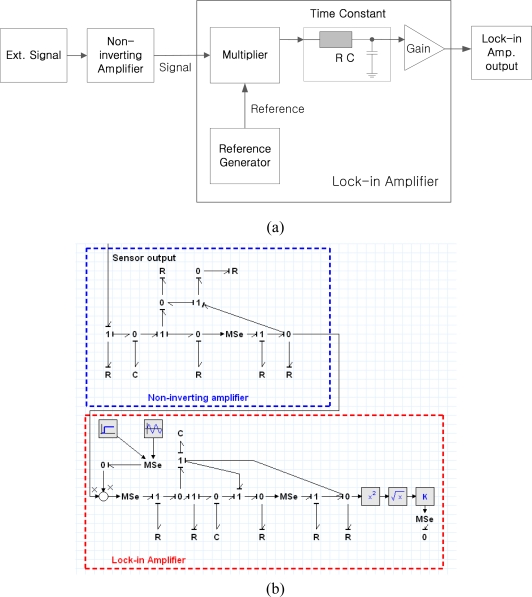
(a) Overall electrical circuit system; (b) Bond graph model of electrical circuit part.

The electrical circuits are mainly composed mainly of electric resistors, capacitors, and operational amplifiers. Resistors and capacitors, which are the passive electrical components, can be expressed with an *R_e_* element and a *C_e_* element. Active electrical components, such as operational amplifier, can be developed in a bond graph model [24]. A noninverting amplifier and a lock-in amplifier can be modeled with standard elements of a bond graph and the developed bond graph model. Figure 15(a) shows an equivalent electrical circuit and lock-in amplifier; Figure. 15(b) shows the bond graph model of an electric system.

### 4.4. System model identification

The numerical values of the electrical and thermal parameters of the system model must be determined for simulation purposes. The values of the passive electrical components are identified from direct measurement. The manufacturer’s data are used to obtain the values of the active electrical component, such as the open loop gain and the input and output resistances values.

Since the heater resistance varies with its temperature, the heat input from the heater continually changes with the resistance of the heater, even if a constant current input is applied. The relation between the resistance of the heater and the temperature is identified as per standard No. 51–1 EIA/JEDEC [31]. The sensor placed in the insulation chamber with the heating element and the heater is connected with the sourcemeter for the purpose of measuring the resistance of the heater. The resistance value of heater and the temperature of insulation chamber at equilibrium, with increasing temperature of insulation chamber by the heating element, are recorded. The relation for this experiment is expressed as:

(4) $$R_{\mathrm{heater}}=124.3\cdot (1-0.0009\cdot T)\;[\Omega ]$$

The thermal parameters of the sensor were identified by means of an analytical calculation and an experimental method. The thermal parameters of the inner parts of the sensor, which consist of the thermal resistance values and the thermal capacitance values of the thermopile and membrane, were determined by numerical calculations based on both the geometry of the sensor and the thermal properties of the materials. The thermal resistance between the surface of the sensor and the ambient is determined experimentally [32]. The power is supplied to the heater in a stepwise manner with the sourcemeter. The temperature response of the sensor surface is measured with an IR thermometer. The thermal resistance is calculated with the measured temperature increment, the ambient temperature, and the measured power dissipation.

### 4.5. Results

**Figure 16.**
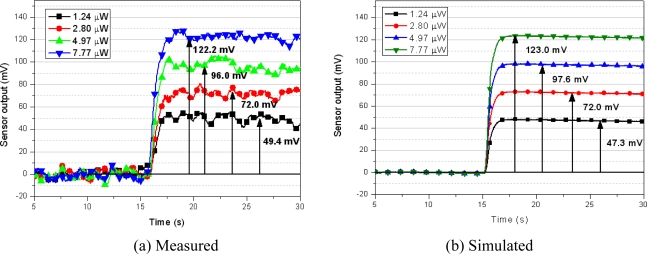
Comparison of the heat-flux output for calibration (input conditions: reference 7 Hz; 265 mV_pp_; 530 mV_Offset_; time constant 300 ms).

The simulation results of the developed system are validated with a comparison of the experimental data. Figure 16(a) shows the measured data, and Figure 16(b) shows the simulated results with different power supplies ranging from 1.24 to 7.77 μW with the sourcemeter.

**Figure 17.**
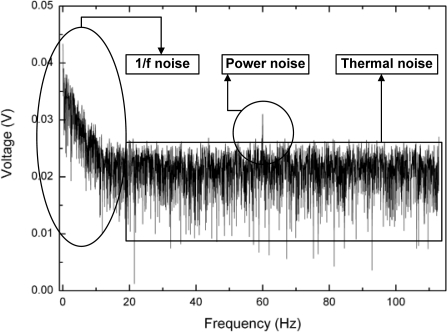
Fourier transform of the sensor output signal.

The experimental data has 20 mV of maximum fluctuations after reaching steady-state. This is possibly arising from the sensor geometry which has long sensitive area and that makes non-uniform temperature on it, but it is not identified yet. To compare between experimental data and simulation data, the sensor output is averaged for 10 seconds after it reaches steady-state. The simulation results follow the measured data for output voltage deviations for less than 4.2%. Figure 17 shows the Fourier transform of the sensor output signal and 20 mV of the dominant noise can be seen.

**Figure 18.**
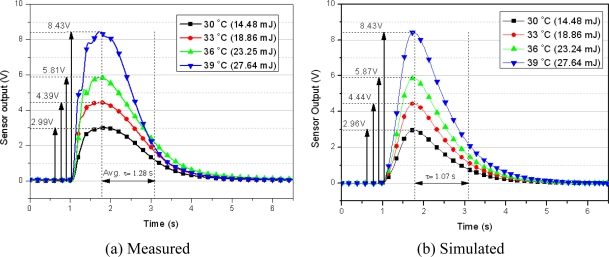
Comparison transient response of the heat-flux output with a fluid injection (input conditions: fluid injection: 350 nL; Ambient temperature: 20.1 °C; reference flow rate: 830 nL/s).

Figure 18(a) shows the measured data, and Figure 18(b) shows the simulated results with a heat supply of 14.48 to 27.64 mJ per 350 nL for the microfluidic application. The simulation results follow the measured data for output voltage deviations of less than 1% and a time constant discrepancy of 0.2 s. As it can be seen, the simulation results are in good agreement with the measured voltage responses. Because the thermal parameters of the inner part of the sensor are calculated on the basis of the geometrical dimensions, there are slight discrepancies between the measured data and the simulation data. If thermal properties of the layers are investigated in terms of the fabrication process of each test sample, the simulation model should be able to produce more accurate results. In the microfluidic application simulation, accurate measurement of the heat and velocity of the fluid can increase the accuracy of the simulation model.

## 5. Conclusions

In this paper, we present the design and fabrication process of a microfluidic heat flux sensor. The microfluidic heat flux sensor system consists of a thermopile, a micro calibration heater, a thermistor, a PDMS fluid channel, and electronic circuits. The sensor was fabricated by a complementary-metal-oxide-semiconductor-compatible process. The characteristics of the sensing system are investigated by means of a thermal modulation method to reduce the low-frequency noise. The system has a sensitivity of 461 V/W and a resolution of 20 nW. The developed microfluidic heat flux sensor was measured with various amounts of fluidic heat to investigate the suitability of the sensor for microfluidic applications. Modeling and simulation of the electro-thermal behavior of the microfluidic heat flux sensor with an integrated electronic circuit and lock-in amplifier are presented for the purpose of designing the micro heat flux sensor system and analyzing the signal output. The proposed system model shows good agreement with the measured data.

## Figures and Tables

**Table 1.** Measurement conditions.

**Modulation condition**	Frequency	7 H_Z_
	Peak to peak voltage	1 V_vv_
	Offset voltage	1 V_Offest_

**Lock-in amp. setting Value**	Time constant	300 ms
	Sensitivity	200 $\mathrm{mV}/\sqrt{\mathrm{Hz}}$
	Expand gain	× 100

## Abbreviations Used

*C*: Capacitance
*R*: Resistance
*T*: Temperature
*T*: Time
*V*: Voltage
*ω*: Frequency
*Φ*: Phase lag

**Subscripts**
AC: AC component
amb: Ambient
DC: DC component
e: Electrical domain
ext: External input
heater: Heater
out: Lock-in amplifier output
sensor: Sensor
th: Thermal domain
ref: Reference component
ref_f: Reference fluid
*ω*: *ω* frequency component
*2ω*: *2ω* frequency component

## References

[1] Diller TE (1993). Advances in heat flux measurement. Advances in Heat Transfer.

[2] Eminoglu S, Sabuncouglu Tezcan D, Yusuf Tanrikulu M, Akin T (2003). Low-cost uncooled infrared detectors in CMOS process. Sensor. Actuator. A-Phys.

[3] Matsumiya M, Shin W, Izu N, Murayama N (2003). Nano-structured thin-film Pt catalyst for thermoelectric hydrogen gas sensor. Sensor. Actuator. B-Chem.

[4] Baciocchi M, Bearzotti A, Gentili M, Lucchesini A (1990). Cu/Pd Thin-film thermopile as a temperature and hydrogen sensor. Sensor. Actuator. A-Phys.

[5] Buchner R, Sosna C, Maiwald M, Benecke W, Lang W (2006). A high-temperature thermopile fabrication process for thermal flow sensors. Sensor Actuator A-Phys.

[6] Kim TH, Kim SJ (2006). Development of a micro-thermal flow sensor with thin-film thermocouples. J. Micromech. Microeng.

[7] Hopper RH (2010). Use of carbon micro-particles for improved infrared temperature measurement of CMOS MEMS devices. Meas. Sci. Technol.

[8] Johannessen EA, Waver JMR, Cobbold PH, Cooper JMA (2002). Suspended membrane nanocalorimeter for ultralow volume bioanalysis. IEEE Trans. Nanobiosci.

[9] Giraldo-Gutierréz L, Moreno-Piraján JC (2005). Determination of the temperature change by means of an outcoming signal of electric resistance in an isoperibolic calorimetric cell. Obtainment of heat solution. Sensors.

[10] Kao PH, Shih PJ, Dai CL, Liu MC (2010). Fabrication and characterization of CMOS-MEMS thermoelectric micro generators. Sensors.

[11] Maskow T, Harms H (2006). Real time insights into bioprocesses using calorimetry: State of the art and potential. Eng. Life Sci.

[12] Macfarlane GG (1950). A Theory of contact noise in semiconductors. Proc. Phys. Soc. B.

[13] Randjelović D, Petropoulos A, Kaltsas G, Stojanović M, Lazić Ž, Djurić Z, Matić M (2008). Multipurpose MEMS thermal sensor based on thermopiles. Sensor. Actuator. A-Phys.

[14] Oh SH, Lee KC, Chun J, Kim MH, Lee SS (2001). Micro heat flux sensor using copper electroplating in SU-8 microstructures. J. Micromech. Microeng.

[15] Oh SH, Lee SH, Jeon JC, Kim MH, Lee SS (2006). Bulk-micromachined circular foil type micro heat-flux sensor. Sensor. Actuator. A-Phys.

[16] Zhang Y, Tadigadapa S (2004). Calorimetric biosensors with integrated microfluidic channels. Biosens. Bioelectron.

[17] Baier V, Födisch R, Ihring A, Kessler E, Lerchner J, Wolf G, Köhler JM, Nietzsch M, Krügel M (2005). Highly sensitive thermopile heat power sensor for micro-fluid calorimetry of biochemical processes. Sensor Actuator A-Phys.

[18] Dehé A, Fricke K, Hartnagel HL (1995). Infrared thermopile sensor based on AlGaAs-GaAs micromachining. Sensor Actuator A-Phys.

[19] Johannessen EA, Weaver JMR, Cobbold PH, Cooper JM (2002). Heat conduction nanocalorimeter for pl-scale single cell measurements. Appl. Phys. Lett.

[20] Kemp RB, Guan Y (1997). Heat flux and the calorimetric-respirometric ratio as measured of catabolic flux in mammalian cells. Thermochim. Acta.

[21] Olmo E (1983). Nucleotype and cell size in vertebrates: A review. Basic Appl. Histochem.

[22] Paynter HM (1961). Analysis and Design of Engineering Systems.

[23] Karnopp D, Rosenberg R (1968). Analysis and Simulation of Multiport Systems.

[24] Granda JJ (2002). The role of bond graph modeling and simulation in mechatronics systems-an integrated software tool: CAMP-G, MATLAB-SIMULINK. Mechatronics.

[25] Thoma JU (1971). Bond graphs for thermal energy transport and entropy flow. J. Franklin Inst-Eng. Appl. Math.

[26] Pal SK, Linkens DA (2002). Temperature distribution in steel during hot rolling: pseudo-bond graph view. Simul. Modell. Practice Theory.

[27] Kim SM, Lee SK (2001). Prediction of thermo-elastic behavior in a spindle-bearing system considering bearing surroundings. Int. J. Machine Tools Manuf.

[28] Kim JK, Nakayama W, Ito Y, Shin SM, Lee SK (2009). Estimation of thermal parameters of the enclosed electronic package system by using dynamic thermal response. Mechatronics.

[29] 20-sim program. Available online: http://.20sim.com/ (accessed on 16 October 2007).

[30] Incropera FP, DeWitt DP, Bergman TL, Lavine AS (2006). Fundamentals of Heat and Mass Transfer.

[31] JEDEC (1995). Integrated Circuits Thermal Measurement Method-Electrical Test Method (Single Semiconductor Device).

[32] Kim JK, Kim TH, Cho SC, Shin SM, Lee SK (2009). Modeling and fabrication of thin film thermopile sensor. J. Vac. Sci. Technol. B.

